# A multiple baseline trial of adapted prolonged exposure psychotherapy for individuals with early phase psychosis, comorbid substance misuse, and a history of adversity: A study protocol

**DOI:** 10.3389/fpsyg.2022.1012776

**Published:** 2022-12-12

**Authors:** Victoria C. Patterson, Philip G. Tibbo, Sherry H. Stewart, Joel Town, Candice E. Crocker, Zenovia Ursuliak, Siranda Lee, Jason Morrison, Sabina Abidi, Kara Dempster, Maria Alexiadis, Neal Henderson, Alissa Pencer

**Affiliations:** ^1^Department of Psychology and Neuroscience, Dalhousie University, Halifax, NS, Canada; ^2^Department of Psychiatry, Dalhousie University, Halifax, NS, Canada; ^3^Mental Health and Addictions, Nova Scotia Health, Halifax, NS, Canada; ^4^Mental Health and Addictions, IWK Health, Halifax, NS, Canada

**Keywords:** prolonged exposure, early phase psychosis, adversity, substance misuse, cognitive-behavioral therapy

## Abstract

**Background:**

Adversity is prevalent among people with psychotic disorders, especially those within the first 5 years of a psychotic disorder, called early phase psychosis. Although adversity can lead to many negative outcomes (e.g., posttraumatic stress symptoms), very few treatments for adversity-related sequelae have been tested with individuals with psychotic disorders, and even fewer studies have specifically tested interventions for people in early phase psychosis. Furthermore, people who misuse substances are commonly excluded from adversity treatment trials, which is problematic given that individuals with early phase psychosis have high rates of substance misuse. For the first time, this trial will examine the outcomes of an adapted 15-session prolonged exposure protocol (i.e., PE+) to observe whether reductions in adversity-related psychopathology occurs among people with early phase psychosis and comorbid substance misuse.

**Methods:**

This study will use a multiple-baseline design with randomization of participants to treatment start time. Participants will complete baseline appointments prior to therapy, engage in assessments between each of the five therapy modules, and complete a series of follow-up appointments 2 months after the completion of therapy. Primary hypothesized outcomes include clinically significant reductions in (1) negative psychotic symptoms measured using the Positive and Negative Syndrome Scale, (2) adversity-related sequelae measured using the Trauma Symptom Checklist-40, and (3) substance use frequency and overall risk score measured with the Alcohol, Smoking, and Substance Involvement Screening Test. We also anticipate that clinically significant reductions in hopelessness and experiential avoidance, measured with the Beck Hopelessness Scale and Brief Experiential Avoidance Questionnaire, the theorized mechanisms of change of PE+, will also be observed. A secondary outcome is a hypothesized improvement in functioning, measured using the Clinical Global Impression and Social and Occupational Functioning Assessment scales.

**Discussion:**

The results of this treatment trial will contribute to the advancement of treatment research for individuals in early phase psychosis who have current substance misuse and a history of adversity, and the findings may provide evidence supporting the use of hopelessness and experiential avoidance as mechanisms of change for this treatment.

**Clinical trial registration:**

Clinicaltrials.gov, NCT04546178; registered August 28, 2020, https://clinicaltrials.gov/ct2/show/NCT04546178?term=NCT04546178&draw=2&rank=1.

## Introduction

### Adversity and substance misuse among people with psychotic disorders

Adversity, which can be defined as the experience of a negative life event that was stressful, uncontrollable, and either was or could have been harmful (Burgermeister, 2007), encompasses both traumatic events (e.g., child abuse) and non-life-threatening events with a similarly negative impact (e.g., discrimination). Adversity exposure is a significant individual influence on the onset of psychosis and clinical outcomes (Janssen et al., 2004; van Os et al., 2009; Conus et al., 2010; Varese et al., 2012). The psychosis proneness-persistence-impairment model (van Os et al., 2009) states that psychological mechanisms, many of which are common outcomes of adversity exposure (e.g., dissociation, external locus of control), can sensitize an individual at risk for psychosis, resulting in the emergence and persistence of psychotic symptoms. Previous studies have found rates of adversity exposure among young adults in early phase psychosis (EPP; i.e., first 5 years of a psychotic illness) ranging from 30 to 96% (Neria et al., 2002; Gearon et al., 2003; Bendall et al., 2007; Üçok and Bıkmaz, 2007; Ramsay et al., 2011; Varese et al., 2012; Trauelsen et al., 2015; DeTore et al., 2021), with a mean of four lifetime adverse event exposures (Gearon et al., 2003; Steel et al., 2011). Adversity exposure is associated with delays in accessing treatment for psychosis (Veru et al., 2022), experiencing more severe psychotic symptoms (Bailey et al., 2018), and a slower recovery during treatment for psychosis (Aas et al., 2016). Experiencing both adversity and EPP is associated with the development of comorbid psychopathology (e.g., depression, post-traumatic stress disorder; Trauelsen et al., 2015), including the development of substance misuse (Phillips and Johnson, 2001; Khoury et al., 2010).

Substance misuse (SM), defined as the problematic use of drugs and alcohol that interferes with functioning, represents another major individual influence on psychosis onset and clinical outcomes (National Collaborating Centre for Mental Health (UK), 2008; Nathan and Lewis, 2021). SM is another broad term that encompasses but is not limited to substance use disorders (SUDs), as well as including substance use that is harmful (e.g., binge drinking) but does not meet criteria for an SUD (Mclellan, 2017). Similar to the proneness-persistence-impairment model above, the stress and coping theory of SM (Wills and Hirky, 1996) posits that psychological mechanisms (e.g., self-efficacy) may play a role in the development and maintenance of SM. Estimates of SM prevalence among individuals with EPP exceed 80% (Ouellet-Plamondon et al., 2017; Cookey et al., 2020), which is remarkably elevated when compared to the 50% prevalence rate among people who have been living with psychosis for over 10 years [i.e., chronic psychosis (Rosenberg et al., 2007)]. Cannabis and alcohol are the most commonly misused substances among people with EPP, with estimated prevalence rates of 70 and 62% (Cookey et al., 2020), and nearly 25% of those in EPP engage in polysubstance misuse (i.e., misuse of 2 or more substances; Ouellet-Plamondon et al., 2017).

SM is associated with more negative outcomes related to the psychotic disorder (Lambert et al., 2005), including increased hallucinations and delusions, lower recovery rates, and lower functioning (González-Pinto et al., 2011; Abdel-Baki et al., 2017). Individuals with SM, psychosis, and a history of adversity also report more distressing hallucinations (Steel et al., 2011), a higher likelihood of developing PTSD (Gearon et al., 2003), and an increased risk of victimization in adulthood (Walsh et al., 2003; Seid et al., 2021). In summary, adversity and SM are highly prevalent among individuals with EPP, they may play a role in psychosis onset, and they are associated with negative outcomes that have a significant impact on the individual level.

### Benefits of adversity-specific treatment in EPP

Psychological treatments may be especially effective for people with EPP, a history of adversity, and SM. This type of treatment can target adversity-related sequelae that trigger and maintain psychosis and SM (e.g., avoidance and dissociation). In addition, treatment can target common comorbid psychopathology (e.g., depression and anxiety) that may be lowering functioning (Scheller-Gilkey et al., 2004), causing distress, and lowering the quality of life.

There is some evidence that psychological interventions targeting adversity-related sequelae delivered to individuals with psychotic disorders may improve long-term outcomes for both psychosis and adversity-related psychopathology (e.g., improved quality of life and increased remission rates; Crumlish et al., 2009; van den Berg et al., 2016), especially for those with a substantial history of adversity (Kilian et al., 2020). Furthermore, compared to individuals with chronic psychosis, young adults in EPP may be able to better engage in and benefit from an adversity-focused psychological intervention because they have not yet sustained the same degree of biological and psychological burden of a long-term psychotic illness (Lieberman et al., 2001).

Importantly, young adults with EPP want treatment for difficulties related to adversity. Australian individuals in EPP discussed their experiences receiving an adversity-focused intervention (Tong et al., 2017), noting that a desire for change was a major motivating factor for participants to initiate and continue to participate in the intervention. Although the participants reported that the intervention was distressing, they also experienced relief and found it beneficial overall (Tong et al., 2017). Participating in an adversity-focused intervention can also help to foster insight into factors leading to the development and maintenance of psychosis (e.g., avoidance), which can aid in recovery (Halpin et al., 2016).

Despite the perceived benefits of participating in an adversity-focused intervention, people with psychosis are routinely excluded; psychosis is the most common exclusion criteria for adversity-specific treatment trials, used in over 90% of trials (Ronconi et al., 2014). Additionally, the few studies that have examined the effects of adversity-focused treatment among people with psychosis primarily focused on individuals with chronic psychosis or included individuals in different phases of a psychotic disorder. Consequently, little is known about treatment effects specifically among people with EPP.

### Adversity-specific treatments for people with psychotic disorders

Steel et al. (2017) conducted a randomized controlled trial (RCT) of cognitive restructuring for posttraumatic stress disorder (PTSD) in individuals with schizophrenia. This treatment did not significantly improve either PTSD or psychotic symptoms—the authors suggested that cognitive restructuring on its own was insufficient and that exposure, an efficacious therapeutic component (see Foa and McLean, 2016, for a review), may be needed to effect clinically significant change. More recently, a trauma-focused CBT for psychosis trial with an exposure component (TF-CBTp; Keen et al., 2017) found that individuals with a psychotic disorder and a complex trauma history experienced improvements in depressive symptoms, anxiety, delusions, PTSD symptoms, and well-being following therapy, although hallucination frequency did not change. Qualitative results highlighted the utility of an integrated approach to treating psychotic symptoms and adversity sequelae. Taken together, these findings suggest that exposure may be needed to effect clinically significant symptom change.

Prolonged Exposure (PE) therapy is an evidence-based form of cognitive behavioral therapy that includes a significant exposure component. PE is one of the most rigorously studied treatment options for people with a psychotic disorder and a history of adversity. An RCT of adults with chronic psychosis and PTSD (mean age = 41) compared PE and EMDR to a waitlist control group (van den Berg et al., 2015). This study found that, compared to the waitlist control group, the PE group experienced a significant reduction in PTSD symptoms and greater rates of PTSD diagnosis remission, even when participants had a dissociative subtype of PTSD (van Minnen et al., 2016). PE therapy also appeared to significantly reduce paranoia and depressive symptoms and improve functioning (de Bont et al., 2016). Grubaugh et al. (2017) replicated these results among veterans with a psychotic disorder and PTSD (*M*_age_ = 46.8). Most participants who completed at least eight PE treatment sessions experienced PTSD symptom remission by the end of treatment. In short, PE therapy appears to effectively reduce psychopathology in individuals with chronic psychosis.

Although some work examines PE treatment among people with psychosis and a history of adversity, there are no PE treatment trials that have included individuals with a psychotic disorder, history of adversity, and SM. In fact, SUDs (previously specified as ‘substance dependence’) are the second most common exclusion criteria for adversity-focused treatment trials, after psychosis, meaning that many individuals with EPP have likely been excluded from previous PE treatment research due to the high rates of substance misuse (a term inclusive of SUDs) among those with EPP. A better understanding of the impact of SM on adversity treatment effects and the effects of adversity-focused treatment on SM may help optimize adversity-focused treatment for individuals with psychotic disorders.

#### Treatments for adversity-related sequelae in people with EPP with SM

Given the existing evidence supporting the efficacy of PE among people with chronic psychosis, adapting a PE protocol for people in EPP with SM may be the optimal path forward. People with EPP are often younger (*M*_age_ = 22.83; Cookey et al., 2020) than those with chronic psychosis (*M*_age_ = 41.2; van den Berg et al., 2015), and people with EPP may be in a better position to benefit from treatment compared to those with chronic psychosis because they have not yet sustained the same degree of biological and psychological burden of substance misuse or a long-term psychotic illness (Lieberman et al., 2001). An adapted PE protocol must be capable of addressing common adverse events experienced by people with EPP (e.g., restraint during hospitalization for psychosis; Carr et al., 2018), accounting for the links between adversity sequelae and both psychosis and SM, and adhering to treatment recommendations for adversity sequelae in EPP. Cragin et al. (2017) interviewed 49 early psychosis treatment experts about suggested clinical treatment guidelines for people with psychotic disorders and comorbid adversity-related sequelae. An integrated treatment approach (i.e., one clinician treating both types of disorders at the same time) was endorsed by experts more often than other possible approaches (e.g., sequenced and parallel). Experts also recommended the following treatment elements: anxiety or stress management, psychoeducation, meditation or mindfulness, cognitive restructuring, interpersonal effectiveness, emotion-focused interventions, and case management. Exposure was rated as a second-line intervention, despite prior evidence that exposure seems necessary for clinically significant symptom change (Taylor et al., 2003; Foa and McLean, 2016). This finding likely speaks to clinicians’ hesitancy to recommend adversity-specific exposure treatments for people with psychotic disorders, given a common fear amongst clinicians of exacerbating psychotic symptoms through exposure (Cragin et al., 2017). More recently, a systematic review of intervention studies for psychotic disorders and trauma (Bloomfield et al., 2020) suggested that future treatments should include many third-wave elements or strategies, such as emotion regulation, psychological acceptance, interpersonal skills, attachment work, strategies to manage dissociation, and trauma memory reprocessing. The review findings indicated that although several studies used an 8-session protocol, future trials should include more sessions to potentially increase the magnitude of treatment effects (van den Berg et al., 2015; Spidel et al., 2019). Overall, the literature supports the use of an integrated treatment approach that uses most core elements of a standard PE protocol with the addition of third-wave strategies and an increased treatment length.

### Aims and hypotheses

The specific aim of this project will be to address the identified treatment gap in early intervention care by applying an adapted PE therapy protocol, called PE+, to a younger EPP population with a history of adversity and current substance misuse. We plan to (1) establish the impact of PE+ on the severity of psychotic symptoms, substance misuse, adversity-related symptoms (e.g., anxiety) and (2) discern whether clinically significant change occurs between sessions 8 and 15, which if true would provide support for the argument that longer treatment duration results in significant symptom change in this cohort. We hypothesize that PE+ treatment will result in clinically significant reductions in (1) negative psychotic symptoms (e.g., anhedonia), (2) adversity-related sequelae (e.g., anxiety and insomnia), and (3) the frequency and quantity of SM, and (4) that all reductions will be maintained by 2-months post-treatment. We also anticipate clinically significant reductions in hopelessness and experiential avoidance, the theorized mechanisms of change of PE+. In terms of secondary outcomes, we hypothesize that participants will experience a global improvement in social and occupational functioning from pre-post PE+ therapy that will be maintained 2 months post-treatment.

## Materials and methods

### Design, randomization, and blinding

This study will use a multiple-baseline design (MBD; Kratochwill et al., 2010), a type of single-case experimental design ideal for stringently examining intervention effects. MBDs are AB designs, meaning they have a baseline (‘A’ phase) and intervention (‘B’ phase), and they do not repeat phases, given that behavioral interventions cannot be rescinded after application. Notably, MBDs temporally stagger intervention start time across participants, thereby creating a control group composed of each participant’s pre-intervention scores. Participants will be randomized to a 2-, 3-, or 4-week baseline condition, thereby staggering the intervention start times; participants will be randomized to a treatment start time using a random sampling/assignment generator.^1^ Randomization is used to increase internal validity and minimize bias by preventing participants from being assigned to a treatment start time based on need or symptom severity, especially given that participants are recruited from an outpatient clinic (Kratochwill, 2010). Randomization order will be delivered using sequentially ordered sealed envelopes that will be opened at the time of randomization. Randomization breakdown is as follows: 2-week delay (40%), 3-week delay (25%), 4-week delay (35%).

### Participants and setting

The study will take place at the Nova Scotia Early Psychosis Program (NSEPP), an early psychosis clinic with approximately 250 active patients that is located within a Canadian academic psychiatric hospital in Halifax, Nova Scotia. Most patients are young adults; the mean age of individuals entering the program is 23 years. Individuals must meet the following criteria to participate in the study:

#### Inclusion criteria

1. Current patient at the NSEPP for the duration of the study;
2. Aged 19–35 years;
3. Diagnosis of a primary psychotic disorder (i.e., schizotypal disorder, delusional disorder, brief psychotic disorder, schizophreniform disorder, schizophrenia, schizoaffective disorder, substance/medication-induced psychotic disorder, other specified schizophrenia spectrum or other psychotic disorder, or unspecified schizophrenia spectrum or other psychotic disorder);
4. Diagnosis of a primary psychotic disorder within the past 5 years; participants must not surpass this 5-year diagnostic window while enrolled in the study;
5. Have experienced 1 or more negative, distressing lifetime adverse events (e.g., child abuse, discrimination) listed on the Trauma and Life Events (TALE) checklist that are currently affecting the participant;
6. At least one score within the “moderate” or “high” risk range for any substance (excluding tobacco products) on the World Health Organization’s Alcohol, Smoking and Substance Involvement Screening Test (WHO ASSIST); and
7. Speaks and understands English.

#### Exclusion criteria

1. Aged 36 and older;
2. Aged 18 and younger;
3. Scoring in the ‘high risk’ range for cocaine use on the WHO ASSIST^2^, suggesting significant misuse;
4. Participant does not speak or understand English;
5. Current involuntary inpatient admission in a hospital or under a Community Treatment Order;
6. Documented, diagnosed intellectual disability; and/or
7. Currently participating in any intervention designed to change substance use or treat adversity-related sequelae (e.g., other clinical trials, psychological therapy).

### Measures

#### Eligibility

The TALE checklist (Carr et al., 2018) is a yes/no scale that asks participants which of the listed events they have experienced in their lifetime (e.g., traumatic entry into care), whether these events occurred more than once, and at what age(s) the event(s) occurred. Additionally, participants will be asked whether any adverse events experienced are currently affecting them in any way and to what degree (0, “Not at all” to 10, “Extremely”). The TALE was created as a measure of adverse events specifically for individuals with psychosis, and psychometrics suggest good test–retest reliability ( r *=* 0.90, *p* < 0.001), adequate convergent validity with the Trauma History Questionnaire ( r = 0.69, *p* < 0.001), and moderate construct validity in terms of correlations with Trauma Symptom Questionnaire outcomes ( r *=* 0.37, *p* = 0.02). The WHO ASSIST (WHO ASSIST Working Group, 2002), an 8-item interview, will be used to measure substance use frequency, urge to use, substance-related difficulties in functioning, and challenges with substance use reduction. Responses are made on a 5-point scale (“Never” to “Daily or almost daily”) and scores can range from 0 to 39 for each substance-specific subscale, with higher scores indicating greater substance misuse. The total score for each substance will be used as an indicator of substance misuse. When used with individuals with first-episode psychosis, the WHO ASSIST was significantly correlated with a measure of alcohol use ( r *=* 0.53, *p* < 0.001) and substance dependence ( r *=* 0.44, *p* < 0.001), and it had appropriate internal consistency ratings for the total score (*M*_Cronbach alpha_ = 0.90) and substance-specific subscales (*M*_Cronbach alpha_ = 0.79, SD = 0.08; Humeniuk et al., 2008; Hides et al., 2009).

#### Primary and secondary outcome measures

##### Primary

The primary outcome measures are psychotic symptoms, adversity-related sequelae, and substance misuse. Adversity-related sequelae is the core outcome we are targeting; however, we are also interested in whether it possible to use an integrated treatment approach that also effects change on both psychotic symptoms and substance misuse. Psychotic symptoms will be measured with the use of the Structured Clinical Interview-Positive and Negative Syndrome Scale (SCI-PANSS; Kay et al., 1987), a semi-structured clinical interview measuring both positive and negative symptoms of psychosis. We will use the total score for each of the positive and negative scales; each total score can range from 7 to 49 with higher scores indicating greater positive or negative symptoms. In an early psychosis sample, the SCI-PANSS positive and negative scales had appropriate internal consistency ( α _Positive scale_ = 0.89; α _Negative scale_ = 0.90). The Trauma Symptom Checklist-40 (TSC-40; Elliott and Briere, 1992) will measure adversity-related sequelae (e.g., depression, insomnia). Response options range from ‘Never’ (0) to ‘Often’ (3). We will use the total score and the subscale scores (i.e., dissociation, anxiety, depression, sleep disturbance, sexual problems, and sexual abuse trauma index). Total scores can range from 0 to 120, with higher scores indicating the presence of greater psychopathology, while subscale score ranges vary by concept. Several studies have used the TSC-40 with people with psychotic disorders (Pec et al., 2014; Spidel et al., 2019) although psychometrics have not been computed with this population. Studies with non-psychosis populations have estimated strong reliability for the TSC-40 total score ( Ω = 0.93; Rizeq et al., 2020). Substance misuse will be measured using the WHO ASSIST, described within the ‘Eligibility measures’ section above.

In addition to the above outcomes, we will also measure changes to hypothesized treatment targets that may function as mechanisms of symptom maintenance: (1) experiential avoidance, and (2) hopelessness. The Brief Experiential Avoidance Questionnaire (BEAQ; Gámez et al., 2014) is a 15-item measure of experiential avoidance; we will use the overall score on this measure as an indicator of avoidance. Response options are on a 6-point scale ranging from ‘Strongly disagree’ (1) to ‘Strongly agree’ (6). Total scores can range from 15 to 90 with higher scores indicating higher experiential avoidance. Across three groups (i.e., students, patients, community), internal consistency was estimated to be good (Mean α = 0.84). Hopelessness will be measured with the 20-item Beck Hopelessness Scale (BHS; Beck et al., 1974). Response options are true/false, and we will use the total score on this measure as an indicator of hopelessness. Scores can range from 0 to 20, with higher scores indicating greater hopelessness. In a chronic psychosis population, BHS total score internal consistency (*α* = 0.85) and subscale internal consistency ( α _Negative expectations_ = 0.84; α _Loss of motivation_ = 0.81) were considered good (Kao et al., 2012).

##### Secondary

Functioning will be measured using the Social and Occupational Functioning Assessment Scale (SOFAS; Morosini et al., 2000), a single-item clinician-reported instrument. Ratings range from ‘Persistent inability to maintain minimal personal hygiene/unable to function without harming self or others or without considerable external support’ (1-10) to ‘Superior functioning in a wide range of activities’ (91-100); lower scores indicate greater impairment in functioning. The Clinical Global Impression—Severity of Illness (CGI-S; Guy, 1976) measures the clinician’s judgement of the severity of the participant’s symptoms of mental illness at this time and the Clinical Global Impression –Improvement of Illness (CGI-I; Elliott and Briere, 1992) measures the clinician’s judgement of the degree of improvement from baseline. The CGI-I and-S will serve as additional measures of functioning that differ from the SOFAS in that they provide global estimates of illness severity and improvement, respectively. We will use the total severity score of the CGI-S, which ranges from ‘Normal, not ill at all’ (1) to ‘Among the most extremely ill’ (7), and the total improvement score of the CGI-I, which ranges from ‘Very much improved’ (1) to ‘Very much worse’ (7). Higher scores indicate more severe symptoms on the CGI-S and symptom worsening on the CGI-I. Symptom measures do not necessarily provide information about impairment, therefore the SOFAS will be used to estimate symptom impairment, and the CGI-S will be used as a global rating of severity, given its holistic view of participant symptoms (i.e., accounts for all symptoms, rather than specific symptom domains).

The PTSD Checklist-5 (PCL-5; Weathers et al., 1993; Price et al., 2016) is a shortened 8-item version of the PCL that will screen for PTSD symptomatology (e.g., intrusive thoughts, negative beliefs) and function as a treatment progress monitoring tool. All items are rated on a 5-point scale ranging from ‘Not at all’ (0) to ‘Extremely” (4), and the total score can range from 0 to 32 with higher scores indicating greater PTSD symptomatology. In a community sample, the total score internal consistency for the 8-item PCL-5 measure was high ( α= 0.90; Price et al., 2016). A recent study of the 20-item version of the PCL-5 (Penney et al., 2021) found that this measure had appropriate psychometrics amongst people with psychosis, although the factor structure did differ amongst this group; no analyses of the psychometrics of the abbreviated 8-item PCL-5 measure have been completed to date with people with psychotic disorders.

A measure of therapeutic alliance, the Session Rating-3 (SRS-3; Duncan et al., 2003), will be administered following each therapy session to account for fluctuations in the therapist-participant relationship on assessment scores. This 4-item assessment tool measures the patient’s perception of the therapeutic relationship, goals and topics covered in session, therapist approach/method, and the therapy session overall for each session. Participants will place the SRS-3 directly in a sealed envelope; therapists will not have access to this information during therapy. Total scores can range from 0 to 40 with higher scores indicating greater therapeutic alliance.

### Intervention

**Figure 1:**
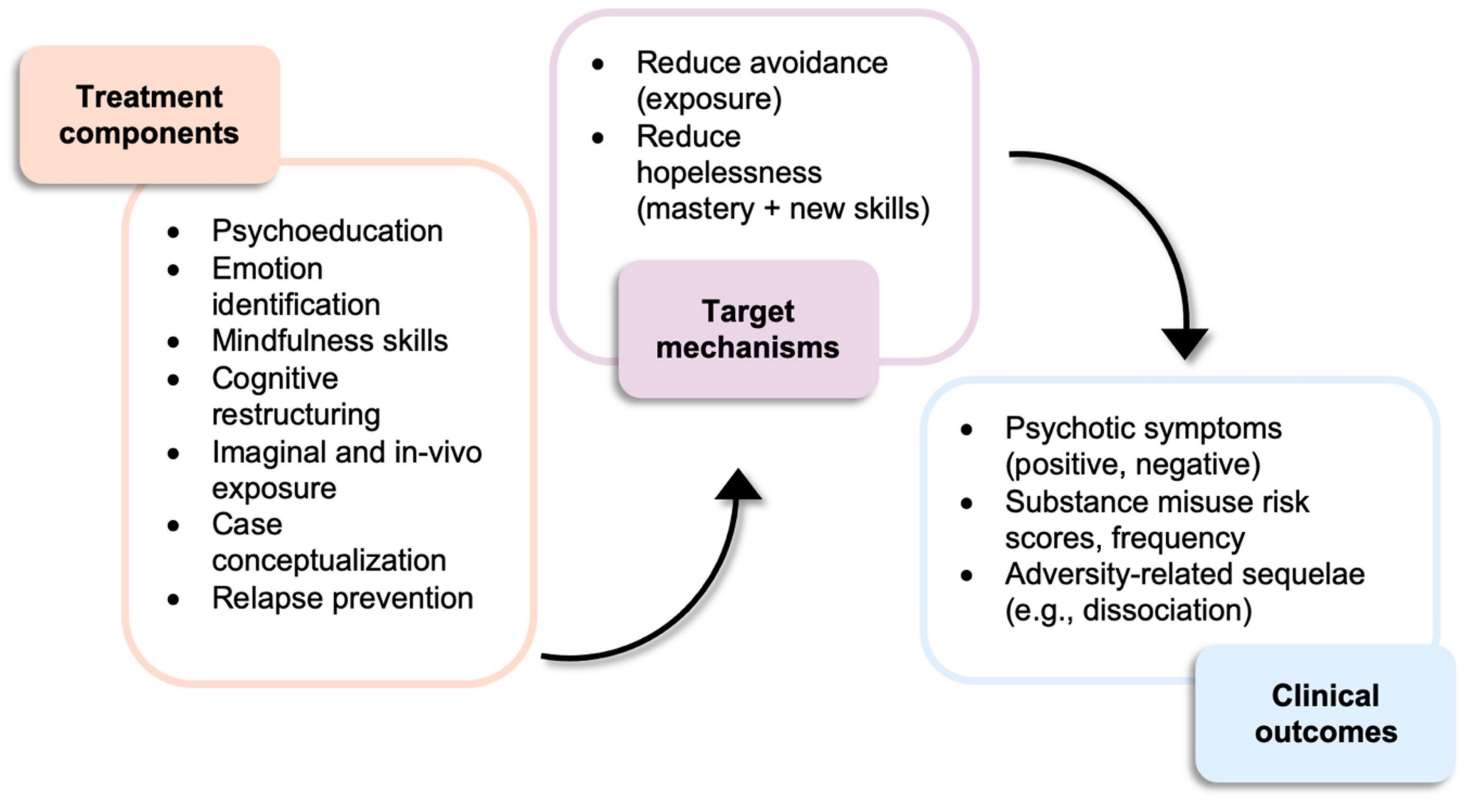
PE+ treatment components, target mechanisms, and clinical outcomes.

This study’s psychotherapeutic intervention, PE+, will consist of a 15-session course of weekly 90-min sessions of adapted PE therapy. The primary theoretical ‘active ingredient’ of PE+ is exposure (i.e., imaginal, *in vivo*; see Figure 1), an effective therapeutic component with substantial evidence supporting its efficacy in treating a variety of mental health challenges, including PTSD and anxiety disorders (see Foa and McLean, 2016, for a review). PE+ uses PE’s theoretical framework, emotional processing theory, which posits that by repeatedly exposing an individual to feared stimuli (e.g., thoughts, feelings, and objects) related to their adverse experience(s), they may generate alternate beliefs and associations with that experience and associated stimuli that may result in a less threatening perspective on the initially feared situation. The American Psychological Association’s (APA) treatment guidelines for CBT therapies for PTSD recommend 4 to 16 sessions of treatment (American Psychological Association, 2017); while fewer sessions might be viewed as more efficient and less costly, several studies testing psychological interventions for adversity-related psychopathology among people with psychosis found that both researchers and participants believed eight sessions was too few (de Bont et al., 2016; Spidel et al., 2019). Therefore, a treatment duration on the longer end of the APA treatment guidelines (i.e., 15 sessions) was selected for the current study.

Treatment will be divided into five modules; each module consists of three sessions. The modules are as follows: (1) psychoeducation about adversity, SM, and the interplay of both with psychosis; (2) emotion identification and regulation; (3) imaginal exposure and identifying thoughts and beliefs, (4) *in vivo* exposures, and (5) planning for termination and maintenance. Module 1 involves an intake interview that includes a suicide risk assessment, followed by psychoeducation about the short and long-term effects of adversity, and the relationship of adversity with psychosis and SM. Psychoeducation will form the foundation upon which the participant can then start to build connections between these experiences within their own life, culminating in a joint case conceptualization at the end of this module. Participants begin discussing their adverse experiences at the end of this first module. Module 2 is focused on aiding participants to develop or enhance their emotional identification and regulation skills, which may help participants effectively process their past experiences. Skills include mindfulness (e.g., nonjudgmental observation), cognitive restructuring (e.g., check the facts), and distress tolerance (i.e., Temperature, Intense exercise, Paced breathing, Paired muscle relaxation) adopted from Dialectical Behavior Therapy (DBT; Linehan, 2014). Modules 3 and 4 are the imaginal and *in vivo* exposure modules. Participants will begin imaginal exposure in the first session of module 3 and *in vivo* exposures will begin the first session of module 4; both types of exposures will continue until the end of treatment (i.e., imaginal exposure across 9 sessions, *in vivo* exposure across 6 sessions). Exposure (i.e., imaginal, *in vivo*) is the core therapeutic ingredient of PE+ treatment, resulting in its greater use across sessions. Imaginal exposures will become more targeted over time to focus on the most difficult moments of past adverse experiences. Module 5 consists of relapse prevention strategies, including identifying helpful aspects of treatment, a final joint case conceptualization, and discussions of preventing symptom relapse. Throughout therapy, participants will be encouraged to practice and further develop the emotional regulation and distress tolerance skills learned in the second module, and participants are asked to listen to recordings of in-session imaginal exposure throughout modules three–five. Homework adherence will be rated at the beginning of every session by participants’ therapist. All session protocols and materials were reviewed and discussed during the design phase of the study with the research team’s patient partner (SL); her expertise was used to modify clinical procedures to improve feasibility for potential participants (e.g., reduction of between-session imaginal exposures).

The study therapists will be three senior PhD students in Clinical Psychology with 3–5 years of clinical experience who have completed training in PE therapy. Training will involve the completion of an online PE certification through PEWeb^3^ and completing and reviewing roleplays of PE treatment elements (e.g., imaginal exposure) as a group over the course of 4 months. Study therapists will be working under the supervision of a clinical psychologist, AP, who has over 20 years of experience providing evidence-based treatment, including CBT for psychosis and substance misuse, and PE for PTSD. Therapists will participate in weekly supervision with AP to discuss session challenges, ethical issues, and treatment fidelity. In addition, study therapists will receive monthly group-based psychodynamic supervision, using video-review of treatment tapes, to identify and formulate participant dissociative processes from an integrative perspective. Prior to delivering treatment, all therapists will complete a two-hour video-based training to supplement supervision. This will be provided by JT, a clinical psychologist with over 15 years of experience and expertise in intensive short-term dynamic psychotherapy (ISTDP) and psychotherapy research. The rationale for the inclusion of this additional training and supervision is the necessity to identify and address dissociative processes as they are occurring as dissociation may interfere with treatment effects. Study therapists will also conduct study assessments, although no therapist will also act as an assessor for the same participant; therapists will be blinded to assessment results during treatment. Any instances of unblinding will be reported in the publication of trial results.

#### Treatment fidelity monitoring

As part of the National Institutes of Health’s (NIH) Behavior Change Consortium, Bellg et al. (2004) outlined a series of strategies to enhance treatment fidelity in treatment studies. These strategies facilitate the five elements of treatment fidelity: (1) treatment adherence, (2) therapist competence, (3) treatment differentiation, (4) treatment receipt, and (5) treatment enactment. We will use the NIH Behavior Change Consortium framework of treatment fidelity to assess treatment fidelity within this trial using both direct (e.g., review of videotaped therapy sessions) and indirect (e.g., questionnaires, adherence checklists) assessment strategies (see Supplementary material for a full description of study treatment fidelity strategies).

We will use a study manual with manualized treatment sessions to ensure equivalent delivery across participants, and therapists will be trained in all treatment and assessment components together to ensure standardized training across clinicians. Therapists will participate in training that includes a significant role-playing and videotape review component to ensure therapist competence is achieved before beginning treatment delivery. Following the completion of all therapy sessions, 10% of therapy session videos will be randomly selected for adherence review by two independent raters experienced in psychotherapy delivery. Video reviewers will use a predetermined checklist of session components to rate videos with each item score ranging from ‘0’ (did not include) to ‘2’ (complete inclusion); session scores must total at least 80% of the total possible score based on the predetermined elements for that session to be considered adherent. There is little agreement in the field about what constitutes an appropriate benchmark for within-session treatment adherence. However, a previous study found that the mean session adherence rate for therapists was approximately 80%, which was considered highly adherent (Huppert et al., 2001). We will adopt a similar standard, especially given that treatment fidelity checklists are detailed, thereby creating a conservative standard for adherence. The video review process will be supervised by a licensed clinical psychologist, AP, who will provide training during this study. In addition, therapists will be provided with weekly supervision, including video review, to minimize therapist drift.

### Procedure

**Figure 2:**
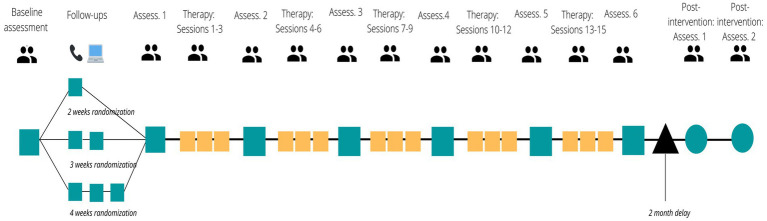
PE+ study procedures.

All new NSEPP patients are routinely asked whether they consent to being contacted for research purposes, with approximately 80% agreeing to be contacted. Patients can self-refer to the study or, with their consent, their NSEPP clinician can refer them. Potential participants will be screened with the WHO ASSIST (Hides et al., 2009; Humeniuk and World Health Organization, 2010) and the Trauma and Life Events checklist (TALE; Carr et al., 2018). See Table 1 for measure information, see Figure 2 for procedure details. If the individual is eligible for the study, they will participate in a consent appointment with study research staff that will involve discussing the study and asking participants to sign an informed consent form, followed by either scheduling their baseline assessment for a future date or completing a baseline appointment immediately following the consent process. Baseline assessments will include four self-report instruments, the BEAQ, BHS, PCL-5, and TSC-40, in addition to several clinician-administered measures, such as the SCI-PANSS, which will be used to assess psychotic symptoms, and the CGI-I and-S, along with the SOFAS, which will assess illness severity, symptom change, and functioning. Demographic information related to participants’ age, gender, race, ethnicity, and sexual orientation will also be collected; these variables are critical to collect as participants from a marginalized community (e.g., 2SLGBTQ+) may have different experiences than those who are not a part of marginalized groups.

**Table 1:** Measures for PE+ study.

Variable	Measure	Items	Timepoints	Report type
Adversity occurrence	TALE Carr et al. (2018)	21	Eligibility assessment, post-therapy follow-up 1	Self-report
Substance misuse	WHO ASSIST WHO ASSIST Working Group (2002)	8	Baseline assessment, Assessments 1–6, Post-therapy follow-ups 1–2	Clinician-administered
Psychotic symptoms	SCI-PANSS Kay et al. (1987)	109^a^	Baseline assessment, Assessment 1, Assessment 6, Post-therapy follow-up 1	Clinician-administered
Adversity-related symptoms	TSC-40 Elliott and Briere (1992)	40	Baseline assessment, Baseline follow-ups 1–3, Assessments 1–6, Post-therapy follow-ups 1–2	Self-report
Experiential avoidance	BEAQ Gámez et al. (2014)	15	Baseline assessment, Baseline follow-ups 1–3, Assessments 1–6, Post-therapy follow-ups 1–2	Self-report
Hopelessness	BHS Beck et al. (1974)	20	Baseline assessment, Baseline follow-ups 1–3, Assessments 1–6, Post-therapy follow-ups 1–2	Self-report
Social and occupational functioning	SOFAS Morosini et al. (2000)	1	Baseline assessment, Baseline follow-ups 1–3, Assessments 1–6, Post-therapy follow-ups 1–2	Clinician report
Illness severity	CGI-S Guy (1976)	1	Baseline assessment, Assessment 1, Assessment 6, Post-therapy follow-up 1	Clinician report
Improvement of illness	CGI-I Guy (1976)	1	Assessment 1, Assessment 6, Post-therapy follow-up 1	Clinician report
PTSD symptoms	PCL-5 American Psychological Association (2017)	8	Baseline assessment, Baseline follow-ups 1–3, Assessments 1–6, Post-therapy follow-ups 1–2	Self-report
Therapeutic alliance	SRS-3 Duncan et al. (2003)	4	Therapy sessions 1–15	Self-report

a Positive and negative SCI-PANSS items only.

This assessment will be followed by 1–3 brief follow-up assessments, depending on the randomization to start time (i.e., 2-, 3-, or 4-week delay between initial interview and therapy) to establish a symptom baseline. The participant’s treatment start time, determined by randomization, will be communicated to the participant at the baseline interview, although the randomization to treatment start time will be communicated to the participant as a part of the consent process. The participant will also participate in an assessment prior to beginning the intervention. The BHS, BEAQ, and TSC-40 will be administered, in addition to the completion of the SOFAS, CGI-I and-S, WHO ASSIST, and SCI-PANSS. After each therapy session, participants will complete the SRS-3 to account for the influence of fluctuations in the therapist-participant relationship on assessment scores, and after each therapy module (i.e., 3 sessions each), current symptoms and SM will be assessed using the instruments above (i.e., BEAQ, BHS, TSC-40, PCL-5, and WHO ASSIST). Psychotic symptoms will be reassessed using the SCI-PANSS after the final session of treatment has been completed. There will also be two follow-up sessions 2-months post-intervention to assess maintenance of therapeutic gains using all the same instruments as at the baseline assessment; each session will take approximately 75 min. Participants will also be asked for their feedback on how to further optimize PE+ therapy for use with patients with EPP in the future and this feedback will be reported and used to optimize this treatment in the future. All participants will be informed that they may discontinue their study participation at any time, and that if psychotic symptoms worsen significantly, they will be referred to their clinician in the early psychosis program for an appointment.

### Data analysis

The goal of this intervention study is to determine the effect of PE+ therapy on psychotic symptoms, substance misuse, adversity-related illness (e.g., PTSD), and functioning. Therefore, the desired outcomes of the analyses will be the significance of symptom change and its maintenance over time. Given the small projected study sample size, inferential statistics are not appropriate. As a result, it is not possible to compute a power analysis; however, a sample of 20 participants is typical for studies using the MBD based on previously published studies using this design (Frueh et al., 2009). Instead of inferential statistics, the Reliable Change Index (RCI; Jacobson and Truax, 1991) will be used to classify participants’ post-intervention score category: recovered (i.e., met criteria for clinical change), improved (i.e., have statistically significant change but not large enough to be considered a full recovery), unchanged (i.e., no change over time), and deteriorated (i.e., significant worsening of symptoms over time). We have calculated the numerical criteria needed to assess symptom change using previously published means and standard deviations of the measures we are using (e.g., SCI-PANSS, TSC-40 scores; see clinical trial registration statistical plan at clinicaltrials.gov). The change criterion being used is moderate, meaning clinically significant change is defined as participants’ post-intervention assessment scores falling between the scores of a healthy population and a mentally ill population. This criterion is the most realistic given that we are aiming to treat a multitude of psychological symptoms rather than a single symptom domain (e.g., PTSD symptoms). We will use the RCI to assess whether clinically significant change occurred in (1) hopelessness and avoidance scores, (2) negative psychotic symptoms (e.g., anhedonia), (3) frequency and quantity of substance misuse, and (4) functioning scores, with gains in all symptom domains maintained at 2 months-post treatment.

## Discussion

The results of this novel adaptation study have the potential to further treatment research by determining whether PE+ contributes to clinically meaningful symptom change for individuals with EPP who are experiencing adversity-related mental health challenges and substance-use related issues.

This study has several strengths. PE has been studied within individuals with psychotic disorders; however, adaptations of treatment for those in EPP have not yet been tested. Furthermore, no previous treatment studies have specifically recruited individuals with comorbid SM and directly measured the effect of PE on SM. The inclusion of SM within this study provides a necessary and novel contribution to the literature, whilst the focus on an EPP population extends the existing body of knowledge of adversity-focused treatment in psychotic disorders. The study intervention will take place within a comprehensive early intervention service with an embedded research program; recruiting participants from this service and delivering the PE+ intervention within an existing clinical setting will help enhance the ‘real-world applicability’ of this study’s results, given that this treatment is meant to be delivered in an early intervention service. Moreover, the integration of this treatment within an existing early intervention service will aid with recruitment by using direct clinician referrals as well as providing a built-in safeguard for participants by allowing follow-up clinical care with clinicians for those participants who may experience psychotic symptom deterioration or relapse. A significant strength of this study is the inclusion of a patient partner on the research team; their experience increased the breadth of the team’s expertise and allowed for the patient perspective when creating the treatment protocol and designing treatment materials. Finally, randomization and comprehensive measures of treatment fidelity will help support the internal validity of the empirical findings of this study.

Despite this study’s many strengths, there will be several limitations to its future findings. There is no requirement for participants to meet criteria for a PTSD diagnosis to receive the PE+ intervention, which introduces variability into the results. Participants must present with substance misuse and a history of adversity and ongoing distress related to the event, but their symptom presentation may vary. This approach was felt to be more appropriate for an initial adaptation of this therapeutic approach. In addition, recruitment processes were not standardized, meaning there may be bias introduced *via* clinician referral. All efforts will be made to approach every eligible person; however, some eligibility criteria are not possible to determine without an interview, therefore some potential eligible participants may be missed.

In conclusion, the results of this study may provide support for the use of an adapted PE protocol to treat adversity-related mental health challenges among individuals with early-phase psychosis and current substance misuse, a common clinical presentation, and provide a tailored treatment option for this group of affected individuals in the future. The use of this treatment may help improve long-term outcomes of individuals within early intervention services, reduce the high burden of comorbid psychopathology, and improve social and occupational functioning within this group. Finally, this trial may provide evidence of the promise of this intervention thereby stimulating further research using larger samples and more rigorous designs (e.g., RCT).

## Ethics statement

The studies involving human participants were reviewed and approved by Nova Scotia Health Research Ethics Board (REB#1025608). The patients/participants provided their written informed consent to participate in this study.

## Author contributions

VP and AP conceptualized the study with significant input from PT. SS and JT helped to refine the direction of the study and contributed to planning data collection and analyses. All other authors contributed to the refinement of the protocol and manuscript. All authors contributed to the article and approved the submitted version.

## Funding

The study is funded by the QEII Foundation through a Translating Research Into Care (TRIC) Level 2 grant [#1025210, awarded 2020] (PT, NH as co-PIs, written by VP). The funding source was not involved in the study design and will not provide input about the study execution, analyses, or interpretation of the results.

## Conflict of interest

The authors declare that the research was conducted in the absence of any commercial or financial relationships that could be construed as a potential conflict of interest.

## Publisher’s note

All claims expressed in this article are solely those of the authors and do not necessarily represent those of their affiliated organizations, or those of the publisher, the editors and the reviewers. Any product that may be evaluated in this article, or claim that may be made by its manufacturer, is not guaranteed or endorsed by the publisher.

## Footnotes

^1^ www.randomizer.org

^2^High cocaine use may be too treatment-interfering and prevent meaningful treatment gains given its significant impact on executive functions (Fernández-Serrano et al., 2010), therefore individuals with high levels of cocaine use are excluded.

^3^ http://pe.musc.edu/

## Abbreviations

2SLGBTQ+: two-spirit, lesbian, gay, bisexual, transgender, queer + individuals
APA: American Psychological Association
BEAQ: brief experiential avoidance questionnaire
BHS: Beck hopelessness scale
CBT: cognitive-behavioral therapy
CGI-I: clinical global impression-improvement of illness
CGI-S: clinical global impression-severity of illness
DBT: dialectical behavior therapy
EMDR: eye movement desensitization and reprocessing therapy
EPP: early phase psychosis
ISTDP: intensive short-term dynamic psychotherapy
MBD: multiple baseline design
NIH: National Institutes of Health
NSEPP: Nova Scotia Early Psychosis Program
PCL-5: posttraumatic stress disorder checklist-5
PE: prolonged exposure therapy
PE+: adapted prolonged exposure therapy
PTSD: posttraumatic stress disorder
RCI: reliable change index
RCT: randomized control trial
SM: substance misuse
SCI-PANSS: structured clinical interview-positive and negative syndrome scale
SOFAS: social and occupational functioning assessment scale
SRS-3: session rating scale-3
TALE: trauma and life events checklist
TF-CBT: trauma-focused cognitive-behavioral therapy
TSC-40: trauma symptom checklist-40
WHO ASSIST: World Health Organization’s Alcohol, Smoking, and Substance Involvement Screening Test
