# Supplementary material for: A multiple baseline trial of adapted prolonged exposure psychotherapy for individuals with early phase psychosis, comorbid substance misuse, and a history of adversity: A study protocol
Source: Front Psychol. 2022 Dec 12;13:1012776. doi: 10.3389/fpsyg.2022.1012776 (PMC9791093; doi:10.3389/fpsyg.2022.1012776)
Supplement: Supplementary file 1 [file Data_Sheet_1.docx]

Supplementary Material

# Treatment fidelity across study phases

## Design

Design as it relates to treatment fidelity means ensuring that the study design allows the testing of connections between the variables of interest and the psychological interventions. In this case, it means delineating the theoretical foundation of PE+ (i.e., emotional processing theory), ensuring that the specific interventions (e.g., imaginal exposure, post-exposure processing) are related to this theory, and the design ensures equivalent intervention dose across participants. Previous work (1) suggested the use of a standardized treatment study manual to facilitate consistency across therapists and provide a model for treatment delivery.

| **Goal** | **Recommended strategies** | **PE+ implementation** |
| --- | --- | --- |
| Ensure same treatment dose | - Provide a fixed number of sessions with fixed duration to all participants | - All participants are asked to participate in 15 sessions, a fixed number of sessions, with a fixed duration of 90 minutes each |
|  | - Record deviations of sessions length | - The length of all sessions is reported within the clinical session note |
|  | - Use a scripted treatment manual | - Each session has an outline and scripted sections to increase similarity between therapists |
|  | - Monitor homework completion | - All homework completion is tracked and quantified within the clinical session note template |
|  | - Provide specialized training to therapists to deal with different types of patients equally | - PE+ therapists were trained by multiple trainers to ensure skills are present to manage different presentations of adversity sequelae |
|  | - Ensure the theoretical foundation of the treatment is clearly delineated and the interventions are connected to the theory | - PE+ is based on the theoretical foundation of Prolonged Exposure (PE), which uses the framework of Emotional Processing Theory (EPT) – this theory is summarized in the PE+ study manual |

## Provider Training

Provider training is important when examining treatment fidelity – equivalent training across therapists ensures consistency across therapeutic sessions and participants. Furthermore, training ensures therapists possess the necessary skills to competently deliver treatment.

| **Goal** | **Recommended strategies** | **PE+ implementation** |
| --- | --- | --- |
| Standardize training | - Train therapists together and use same instructors for all therapists | - All therapists were trained together when learning PE+ -related interventions (e.g., imaginal exposures) from Dr. Pencer, a registered psychologist, and how to assess and intervene when participants are displaying dissociative phenomena from Dr. Town, a registered psychologist |
|  | - Use standardized training manuals/materials/provider resources | - The study manual contains standardized session plans, handouts, and resource documents |
|  | - Use structured practice and role-playing | - Therapists engaged in a biweekly clinical skills practice session, which included role-plays to practice specific clinical skills (e.g., post-session processing) and mock assessment scoring over the course of 5 months |
|  | - Design training to allow for diverse implementation styles and experience levels of therapists | - Therapists are all PhD students in Clinical Psychology and training accounted for all levels of experience and study procedures allow for some flexibility in implementation style |
| Ensure provider skill acquisition | - Score provider adherence according to an a priori checklist | - All sessions will be scored immediately after the session by the therapist to establish whether all elements were fully completed, partially completed, or not completed and why. An a priori cut-off score of 80% of each session’s total will be used to determine whether a session was considered acceptably adherent to the session protocol |
| Minimize “drift” in provider skills | - Conduct weekly supervision | - Group supervision will be offered on a weekly basis to therapists to troubleshoot challenges, review case conceptualization, and answer questions |
|  | - Allow therapists easy access to project staff for questions about the intervention | - Group supervisor is also co-PI, and PI is one of the therapists, meaning therapists have easy access to both individuals for questions regarding the intervention and study procedures |
| Accommodate therapists’ differences | - Monitor differential drop-out rates | - Drop-outs will be examined by randomization status (i.e., 2-,3-, or 4-week delay) and provider assignment (i.e., to which therapist they are assigned) |
|  | - Use regular debriefing meetings | - Debriefing meetings will be available to therapists when needed |

## Delivery of Treatment

| **Goal** | **Recommended strategies** | **PE+ implementation** |
| --- | --- | --- |
| Control for provider differences | - Assess participants’ perceptions of provider warmth and credibility via self-report questionnaire and provide feedback to interventionist and include in analyses | - Therapists are evaluated by their clients following each session using the Session Rating Scale 3 (SRS-3) – this measure examines the therapeutic relationship, sessions goals and topics, approach/method, and provides an overall rating. Therapists do not review this feedback during the study to minimize participant bias, however, participants are asked to give their therapists feedback midway through the course of therapy. |
|  | - Conduct a qualitative interview at end of study | - Participants are asked about their perception of their therapist by their assessor following the final treatment session – they are also asked to discuss their perception of what their therapist did that was helpful and unhelpful |
| Reduce differences within treatment | - Use a scripted intervention protocol and treatment manual | - PE+ manual is always accessible to therapists and all sessions have a clear outline and scripted sections |
| Ensure adherence to treatment protocol | - Randomly monitor audiotapes for both protocol adherence and nonspecific treatment effects | - Following the completion of all therapy sessions, 10% of videos will be randomly selected for adherence review   - Two (2) independent reviewers, one of whom will be Victoria Patterson (study PI) and one will be another clinical psychology PhD student trained in Prolonged Exposure, will review a random sample consisting of 10% of the projected ~300 videos (i.e., 30 videos amounting to 45 hours total); several videos from each therapist will be reviewed. VP will not review her own therapy session tapes to minimize bias; all other videos will be double rated.   - The two raters will review videos against a predetermined checklist of session elements – scores include ‘did not include (0)’, ‘partial inclusion (1)’, and ‘complete inclusion (2).’ Session scores must total at least 80% of the total possible score based on the predetermined elements for that session to be considered adherent (e.g., a total score of 16 out of 20 is considered sufficiently adherent, whereas 14 of 20 is not).   - This rating procedure will be supervised by Dr. Pencer, an expert in psychotherapy, psychosis, adversity, and substance misuse |
|  | - After each encounter, have provider complete a behavioural checklist of intervention components delivered | - Adherence checklists are filled out by therapists following each session, which also enables the reporting of session protocol deviations |
| Minimize contamination between conditions | - Use treatment-specific handouts, presentation materials, manuals | - All handouts, materials, and the manual are treatment-specific to PE+ |
|  | - Supervise therapists frequently | - Therapists receive weekly supervision |

#### Receipt of Treatment

The ability to understand treatment information and skills represents an important element of treatment fidelity. This element allows participants to effectively utilize information gained and permits an accurate measure of fidelity – participants cannot use information they do not understand. Within this study, measuring treatment receipt means checking for comprehension, tracking homework completion, and using strategies to maximize comprehension (e.g., summarizing).

| **Goal** | **Recommended strategies** | **PE+ implementation** |
| --- | --- | --- |
| Ensure participant comprehension/participant ability to use cognitive skills | - Have therapists review homework or self-monitoring logs | - Therapists review homework tasks near the beginning of each session to solidify learning, correct any misconceptions or misunderstandings, and scaffold content. In addition, homework completion is tracked to help measure adherence as an indirect measurement of comprehension |
|  | - Use scripts that prompt therapists to paraphrase/summarize content | - Every session begins with a summary of the previous session’s content, and paraphrasing/summarizing is encouraged throughout treatment to facilitate learning and build rapport |
|  | - Have therapists monitor and give feedback on practice sessions | - Participants are taught a series of emotion-focused coping skills throughout the early modules of the treatment, and therapists are instructed to provide feedback on skill utilization to maximize effectiveness. |

#### Enactment of Treatment Skills

Enactment scaffolds well onto treatment receipt – participants may understand the information and skills learned, but if they are not used, treatment will minimally affect behaviour and/or outcomes. Tracking the extent of use of treatment skills provides information on treatment enactment.

| **Goal** | **Recommended strategies** | **PE+ implementation** |
| --- | --- | --- |
| Ensure participant use of cognitive skills | - Use self-report regarding achievement of goals | - The post-therapy feedback questionnaire includes items such as “How helpful was this therapy in helping you achieve the goals you set at the beginning of treatment?” and follows a discussion of goal review during the final module of therapy |
|  | - Discuss ongoing use of skills with participants | - Participants will discuss their skill use throughout treatment with their therapist and troubleshoot issues throughout to minimize barriers to skill use |
| Ensure participant use of behavioural skills | - Monitor frequency of sessions | - Session frequency is recorded, as are deviations from the study protocol delineating a schedule of weekly therapy appointments |
|  | - Observe in vivo interactions | - Therapists will observe participants’ familiarity with treatment skills to establish use |
|  | - Assess skill use with questionnaires | - Participants will complete a feedback measure post-therapy that asks about frequency of use of skills learned/used in treatment |

References

1. Kaderavek JN, Justice LM. Fidelity: An essential component of evidence-based practice in speech-language pathology. American Journal of Speech-Language Pathology. 2010;19(4):369–79.
